# Periodic Vomiting with Acetonæmia in Children

**Published:** 1906-03-24

**Authors:** 


### PERIODIC VOMITING WITH ACETONÆMIA IN CHILDREN.

Under the above heading Dr. Vincent Dickinson describes, in the current number of the "British Journal of Children's Diseases," a form of vomiting which has received a good deal of attention in France and Italy, but not in this country. The clinical picture of the malady as given by the author may be abbreviated as follows: "In the midst of perfect health, without apparent or appreciable cause, or error in diet, or derangement of intestinal action, the child, who is usually of a nervous or arthritic diathesis, is suddenly seized with vomiting. The vomiting is produced by regurgitation without any premonitory nausea, just as in cases of tuberculous meningitis, the vomit being usually watery, sometimes alimentary or bilious; but when this latter is noticed, which is not often, it indicates that the end of the atack is approaching. Attacks of this kind of vomiting happen several times a day, and recur with variable frequency, sometimes every quarter of an hour. The whole attack lasts usually from two to four or five days, but is sometimes prolonged for two or even three weeks, it being impossible to predict the moment when it will come to an end. Once at an end it leaves no after-effect, and health is re-established without convalescence. After a variable interval of time, a few days to a few months, the attacks of vomiting are renewed with the same symptoms, and the illness may thus continue for years, disappearing completely about the time of puberty.

"The symptoms which are associated with the vomiting are variable. Usually at the onset of the attack the child is prostrate, lies motionless in bed, neither crying nor groaning, its face pale and drawn, the eyes sunken and the abdomen retracted; very often there is constipation, rarely diarrhœa, the tongue is relatively clean, the pulse almost always accelerated, there may be also irregularity. The temperature does not regularly exceed 100.4° F., very rarely reaching 102.4° F. In exceptional cases it remains normal. Gastric pain is almost always absent, a point of diagnostic importance; when it does occur it is never at the commencement, but only at the end of the attack, and even then the pain only appears at the moment of vomiting, and ceases in the interval. Thirst is intense, but the child cannot drink, for the least quantity of fluid is sufficient to provoke a fresh attack. The appetite is always retained, an important diagnostic point. To recapitulate, the abrupt onset, the absence of previous gastro-intestinal disturbance, the absence of anorexia, the absence of gastric pain, the character of the vomiting, the slight elevation of temperature, these are the most important signs to be considered in arriving at a clear conception of the affection. During the attack the breath has a distinct smell of acetone, so marked as to be noticeable by anyone entering the sick-room. The urine is scanty and, on analysis, shows a definite amount of acetone, and often also of indican."

A great variety of hypotheses attempts to meet this condition. Comby considers it a manifestation of the arthritic diathesis, because the parents of the sufferers are generally "obese, gouty, or eczematous." Snow and Whitney call it a gastric neurosis. Edsall considers it "a grave acid-intoxication of a type frequently met with in diabetes mellitus, an intoxication whose origin cannot be precisely defined." While unacquainted with the condition ourselves, we may observe that unless the smell of acetone is very marked the diagnosis from tuberculous meningitis will be almost impossible. It is true that, as the author remarks, in tuberculous meningitis there is usually a prodromal stage, but quite often there is not; also, it is barely correct to say that in tuberculous meningitis the vomiting is present only at the beginning of the disease. Relatively to the total duration of the disease this is generally true, but the vomiting of tuberculous meningitis may easily outlast the average span of this "periodic" disturbance. The differential diagnosis assumes an immense importance from the circumstance that while in the case of tuberculous meningitis the outlook is practically hopeless, the periodic vomiting under notice almost always eventuates favourably.

